# Sarcomatoid Urothelial Carcinoma: A Single Cancer Center Experience

**DOI:** 10.4021/wjon370w

**Published:** 2011-08-24

**Authors:** Jue Wang, Caitlynn Gillaspie, Rajesh Kunadharaju, Geoffrey A. Talmon, Charles Enke

**Affiliations:** a Department of Internal Medicine, Section of Oncology-Hematology, University of Nebraska Medical Center, Omaha, Nebraska 68198-7680, USA; b Department of Pathology and Microbiology, University of Nebraska Medical Center, Omaha, Nebraska 68198-3135, USA; c Department of Radiation Oncology, University of Nebraska Medical Center, Omaha, NE 68198, USA

**Keywords:** Sarcomatoid bladder cancer, Cystectomy, Multimodality therapy, Outcome

## Abstract

**Background:**

Sarcomatoid bladder cancer is a rare and aggressive variant of urothelial carcinoma.

**Methods:**

A retrospective review of our experience in managing patients with sarcomatoid bladder cancer (SRBC) between 1997 and 2011 was performed to better define the behavior and outcomes of this disease.

**Results:**

The median age of the patients was 63 years. All patients presented with high grade histology. Eighty-five percent of the patients presented with muscle invasive disease and fifty percent presented with stage IV carcinoma. Ten of 14 (71%) of patients underwent a cystectomy. Patients with SRBC was younger (P < 0.01), more commonly presented with higher grade histology (P < 0.01) and advanced stage disease (P < 0.01), in comparison with patients with Urothelial carcinoma (UC). At a median follow-up of 7 months (range 1.3 - 112), five (35.7%) patients have died in last follow-up. Two-year survival was 53.5%. Three patients with long term survival were reported.

**Conclusions:**

Sarcomatoid bladder cancer is associated with poor prognosis. Multimodality therapy may improve these patients outcome.

## Introduction

Sarcomatoid bladder carcinoma is a rare variant of urothelial bladder cancer [1], and accounts for less than 1% of all urothelial tumors. It is associated with a rapid growth rate and an advanced stage at presentation [1, 2]. Because of the rarity of carcinosarcoma of the urinary bladder, previously published information has been based on case series and anecdotal experiences with a focus on histopathological evaluation [3-12]. However, there are limited information regarding treatment and outcome for this disease [13-16]. The objective of this study was to analyze 14 consecutive cases of patients with muscle-invasive or metastatic sarcomatoid bladder cancer who were treated at a single Cancer Center between 1997 and 2011. This retrospective analysis was carried out in order to gain more understanding regarding the clinical behavior, treatment and outcome of this aggressive disease.

## Methods

### Patient and tumor characteristics

We retrospectively reviewed the medical records of all patients with sarcomatoid bladder carcinoma treated at our cancer center between 1997 and 2011. Patients were considered to have sarcomatoid disease if the pathology report revealed any sarcomatoid component in their tumor. Patient medical records were analyzed for demographic characteristics, clinical stage and outcome. Radiology, pathology and surgical reports were reviewed to determine the pathological staging at the time of cystectomy using the 1997 TNM classification for genitourinary tumors. Chemotherapy regimens, radiotherapy doses, and surgical modality were also recorded. To compare the clinical characteristics and outcomes of patients with sarcomatoid components to those who did not have these components, patients who had sarcomatoid components were matched to patients in bladder cancer database who had no sarcomatoid elements present. The study was approved by the Institutional Review Board of the University of Nebraska, and individual consent for the study was waived.

### Statistical analysis

Descriptive statistics, such as frequency counts, medians, and ranges, were used to characterize the patient sample. Overall survival was calculated from the date of diagnosis to the date of death or the date of last follow-up. Estimation of survival curves was performed using the Kaplan-Meier technique [11]. All statistical calculations were performed by SPSS 12.0 (Apache Software Foundation 2000).

## Results

### Characteristics of patients with sarcomatoid bladder cancer

Details of patient and tumor characteristics of study cohort are included in Table 1 and 2.

**Table 1:** Characteristics of Patients of the Urinary Bladder According Histology

Characteristics	UC (N = 319) (%)	Sarcomatoid (N = 14) (%)	P-value
Age			
Median (Range)	71 (32 - 92)	63 (45 - 93)	
Gender			
Male	243 (76.2)	12 (85.7)	0.53
Female	76 (23.8)	2 (14.3)	
Race			
White	301 (94.0)	14 (100.0)	0.45
Black	18 (6.0)	0 (0.0)	
Grade			
High Grade	182 (57.1)	14 (100.0)	< 0.01
Low Grade	123 (38.6)	0 (0.0)	
Not Specified	14 (4.3)	0 (0.0)	
AJCC Stage			
< T2	182 (57.1)	2 (14.3)	< 0.01
≥ T2	135 (42.3)	12 (85.7)	
TX	2 (0.06)	0 (0.0)	
Year of Diagnosis			
2000-2005	112 (35.1)	8 (57.1)	0.09
2006-2011	207 (64.9)	6 (42.9)	
CDS			
Cystectomy	85 (26.6)	10 (71.4)	< 0.01
TURBT alone	222 (69.6)	4 (28.6)	
None	12 (3.8)	0 (0.0)	

CDS: Cancer Directed Surgery; UC: urothelial carcinoma

**Table 2:** Characteristics of 14 Cases of Patients With Sarcomatoid of the Urinary Bladder

Case	Stage	Tumor Size (cm)	Hydronephoresis	Hematuria	Smoking	Therapy
1	T1N-	4	yes	yes	neg	Cystectomy, TURBT
2	T2N-		no	yes	neg	Cystectomy, TURBT
3	T2N-	2.6	no	yes	pos	Cystectomy, TURBT
4	T4N-	0.6	yes	yes	pos	Cystectomy, TURBT
5	T3N-	2.5	yes	yes	pos	Cystectomy, TURBT
7	T2N+		yes	yes	pos	Cystectomy, TURBT
8	T2N+	8	yes	yes	pos	TURBT, Chemotherapy
9	T1N-	3	no	yes	neg	Cystectomy, TURBT
10	T3N+	4.5	yes	yes	pos	TURBT, Neoadjuvant Chemotherapy, Palliative Radiation Therapy
11	T3N+	7	yes	yes	pos	Cystectomy, TURBT
12	T2N+		yes	yes	neg	TURBT, Chemotherapy
13	T3N+	7	yes	yes	pos	Nephroureterectomy, Adjuvant Chemotherapy
14	T3N+	4.5	yes	yes	pos	TURBT, Chemotherapy
15	T2N-	4.7	yes	yes	neg	TURBT, Radiation therapy

Between January 1997 and April 2011, fourteen patients had sarcomatoid features were treated at our cancer center. Median age at diagnosis was 63 years (45 to 93 years) and male/female ratio was 6:1. Nine of 14 patients (64%) of patients had previous smoking history. All patients presented with hematuria. Eleven of 14 (78.5%) patients presented with hydronephorosis. Twelve of 14 (86%) patients presented with muscle invasive disease and 7 of 14 (50%) with lymph node positive disease. The median tumor size was 4.5 cm (range 0.6 - 8 cm). All the tumors presented with high grade histology.

To compare the clinical characteristics of patients with sarcomatoid components to those who did not have these components, patients who had sarcomatoid components were matched to 319 patients with conventional urothelial carcinoma (UC) of bladder who were treated at same period of time. Table 1 lists demographic and pathological characteristics of the two histologies. Patients with SRBC were younger at diagnosis compared to UC (median age: SRBC 63 versus UC 71). The patients with SRBC presented at more advanced stage than those with UC, as shown by a higher rate of muscle invasive disease (85.7% versus 42.3%, P < 0.01). Higher grade disease (poorly-differentiated or undifferentiated histology) was more common in patients with SRBC (100% versus 57.1%, P < 0.01). Surgery (radical or partial cystectomy) was performed for 71.4% of SRBC and 26.6% of UC cases (P < 0.001).

### Survival

**Figure 1:**
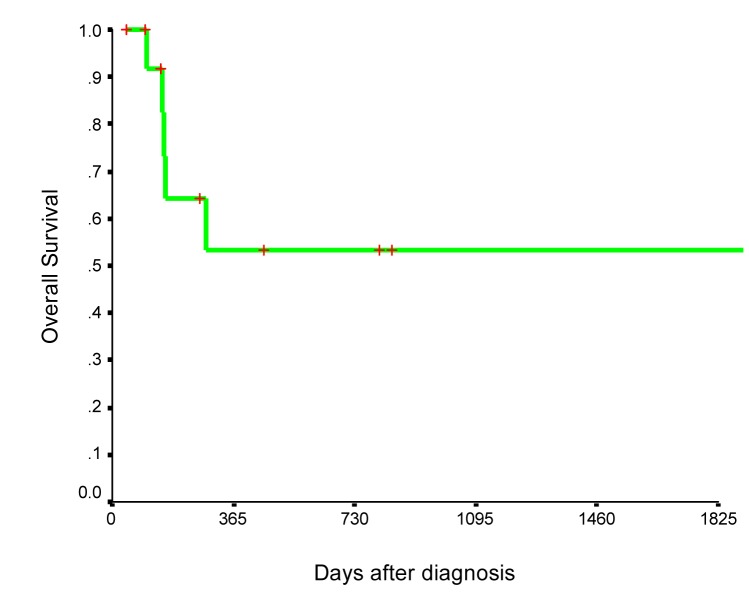
Overall survival of 14 patients with sarcomatoid bladder cancer.

**Figure 2:**
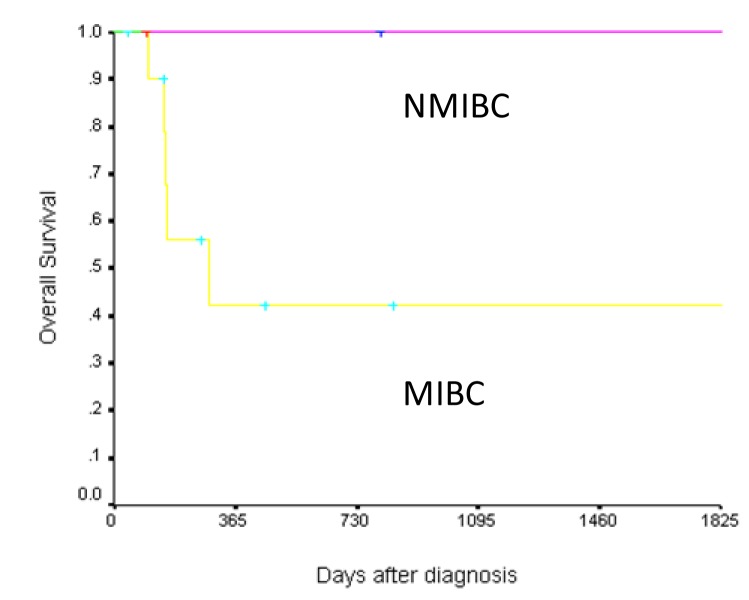
Overall survival of 14 patients with sarcomatoid bladder cancer according to stage (NMIBC: Nonmuscle invasive bladder cancer; MIBC: Muscle invasive bladder cancer).

At a median follow-up of 7 months (range 1.3 - 112), five (35.7%) patients have died in last follow-up. Overall survival is illustrated in Figure 1. The two-year survival rate of the cohort was 53.5%. Figure 2 shows Kaplan-Meier curve of patients with sarcomatoid bladder cancer according to tumor stage.

### Long term survivors

A 76-year-old gentleman presented on May 11, 2001 for gross hematuria. A cystoscopy revealed a large bladder tumor obscuring the right ureteral orifice. The patient had a TURBT and a right ureteral stent placement. The surgical specimen contained for primarily low grade (Grade II/IV) papillary urothelial carcinoma with areas contained sarcomatous and osteosarcomatous differentiation. The patient was subsequently admitted and underwent a bilateral pelvic lymph node dissection, partial cystectomy with distal right ureterectomy, ureteral reimplantation, and placement of a right double-J ureteral stent. The surgical pathology revealed no residual tumor, and five lymph nodes were examined without tumor involvement. The patient remains well with no evidence of recurrence since his last follow-up on August 13, 2010.

A 57-year-old man was evaluated for gross hematuria in May 2008. A cystoscopy and TURP revealed high-grade urothelial carcinoma with sarcomatous involvement. A low-grade noninvasive papillary urothelial bladder neck cancer was also identified. He was treated with a prostatectomy and partial cystectomy, but the surgical margins were positive at the vesical neck. Furthermore, there was extracapsular extension of the tumor. A radical cystectomy was recommended. There was evidence of progression of pelvic lymphadenopathy on serial CT scans, as he considered the procedure over a 6-month period. Eventually, he had a radical cystectomy in March 2009. Surgical pathology revealed one left pelvic node and three right pelvic nodes were positive, and all had extracapsular extension. The bladder remnants were free of disease. The patient was treated with chemoradiation using docetaxel as a radiosensitizer, followed by four cycles of gemcitabine and cisplatin. The patient remains disease free with no evidence of recurrence to date after his last follow-up on July 13, 2011.

A 58-year-old female presented in March 2005 with a two month history of hematuria and increased urinary frequency. The patient underwent a cystoscopy and a TURBT due to a high-grade, muscle-invasive urothelial carcinoma. An anterior pelvic exoneration was planned, and she sought a second opinion at our hospital. A CT scan showed hydronephrosis as well as enlarged pelvic and retroperitoneal lymph nodes. A retroperitoneal lymph node biopsy showed a high-grade malignant tumor with sarcomatoid features consistent with metastatic bladder cancer. The patient was treated with cisplatin and gemcitabine for 4 cycles. Her therapy was completed in April 2005. She has been followed expectantly since then with no evidence of recurrence six years after the diagnosis. She had a durable, complete remission after four cycles of gemcitabine and cisplatin.

## Discussion

The present study showed patients with this rare form of urothelial cancer are diagnosed at a younger age and present with a higher grade of histologic malignancy as well as an advanced stage when compared to patients without sarcomatoid differentiation.

Consistent with previous studies, the cancer specific survival of this cohort of carcinosarcoma of the urinary bladder was poor. Due to the absence of randomized and controlled trials, there is no standard treatment for this disease. Only a few studies reported the use of chemoradiotherapy and chemotherapy after surgical resection of carcinosarcoma of the urinary bladder. In our series, aggressive, multi-modality treatment, in 3 patients, led to complete responses and markedly improved survival. It is gratifying to report the long-term survival, now multiple years, of these three patients with sarcomatoid bladder cancer.

### Conclusion

The low incidence of this variant renders the conduct of randomized trials rather impossible and drawing clear guidelines for its management is subsequently difficult. Nevertheless, our series suggest that long term survival is possible in patients treated with multimodality therapy, and the optimal treatment modality has yet to be defined. Future studies will investigate the combination of chemotherapy with new targeted therapies.
